# Stimulation of carbon nanomaterials on syntrophic oxidation of butyrate in sediment enrichments and a defined coculture

**DOI:** 10.1038/s41598-018-30745-7

**Published:** 2018-08-15

**Authors:** Wei Zhang, Jianchao Zhang, Yahai Lu

**Affiliations:** 1 College of Urban and Environmental Sciences, Peking University, Beijing, 100871 China; 2 Institute of Surface-Earth System Science, Tianjin University, Tianjin, 300072 China

## Abstract

It remains elusive if direct interspecies electron transfer (DIET) occurs in canonical syntrophy involving short-chain fatty acids oxidation. In the present study, we determined the effects of carbon nanomaterials on syntrophic oxidation of butyrate in two lake sediment enrichments and a defined coculture comprising *Syntrophomonas wolfei* and *Methanococcus Maripaludis*. After four continuous transfers of enrichment cultivation, *Syntrophomonas* dominated the bacterial populations in enrichments, and the dominated methanogens comprised *Methanosarcina* and *Methanospirillum* in one enrichment (from Weiming Lake) and *Methanoregula* and *Methanospirillum* in another (from Erhai Lake). Butyrate oxidation and CH_4_ production was significantly accelerated by carbon nanotubes (CNTs) in both enrichments. Replacement of CNTs by magnetite caused similar stimulating effect. For the defined coculture, two carbon nanomaterials, CNTs and reduced graphene oxide (rGO), were tested, both showed consistently stimulating effects on butyrate oxidation. Addition of kaolinite, an electric nonconductive clay mineral, however, revealed no effect. The test on *M*. *maripaludis* in pure culture showed no effect by rGO and a negative effect by CNTs (especially at a high concentration). Fluorescence *in situ* hybridization (FISH) and scanning electron microscopy (SEM) revealed that microbial cells were interwoven by CNTs forming cell-CNT mixture aggregates, and in case of rGO, cells were attached to surface or wrapped-up by rGO thin sheets. Collectively, our data suggest that the presence of conductive nanomaterials likely induces DIET in syntrophic butyrate oxidation.

## Introduction

Microorganisms in nature interact each other and form complicated network. One such specific interaction is syntrophy, a thermodynamically-based cooperation, in which syntrophic partners rely on interspecies electron transfer (IET) to share the minimum free energy for growth^1–3^. These interactions play pivotal role in the degradation of organic matter in anoxic habitats^4^. Fermentation of organic matter produces various intermediate products such as short-chain fatty acids and alcohols, which are chemically more reduced than their precursors^5,6^. The degradation of these intermediates under methanogenic conditions requires tight cooperation between syntrophic bacteria that discharge electrons (oxidation) and produce acetate from intermediates and methanogens that utilize electrons to reduce CO_2_ to CH_4_ and dismutate acetate to CH_4_ and CO_2_. The oxidation step of this process is endergonic which relies on the consumption of products by methanogens to a sufficiently low level to make the reaction thermodynamically feasible. As such, syntrophy represents an energy-specific model of microbial mutualism in nature.

The mechanism how syntrophic organisms coordinate their electron transfer and make growth under energy limitation conditions remains unclear. Different pathways are involved in electron release from oxidation of intermediates. The final sink of the released electrons can be hydrogen and formate, as multiple hydrogenases and formate dehydrogenases, either membrane bound or cytoplasmic, are present in syntrophic organisms^7–9^. However, electron discharge to low potential acceptors (H^+^ and CO_2_) is thermodynamically problematic. To solve the energetic dilemma, reverse electron transfer and flavin-based electron confurcation has been proposed^7,8,10,11^. Reverse electron transfer that processes with the cost of proton motive force is considered to be associated with the membrane-bound externally-oriented hydrogenases and formate dehydrogenases. Cytoplasmic reoxidation of NADH to H_2_/formate occurs likely via electron confurcation by coupling to the oxidation of reduced ferredoxin (Fd_red_). But this idea has been questioned in a recent study showing that reoxidation of NADH by a recombinant hydrogenase from *Syntrophomonas wolfei* did not need Fd_red_^12^.

While clouds still remain on the mechanism of electron discharging via H_2_ or formate by syntrophic bacteria, a new pathway has been disclosed. In a lab-constructed defined coculture of two *Geobacter* species, direct interspecies electron transfer (DIET) was demonstrated independent of H_2_ and formate^13^. Electrically conductive pili (e-pili according to Lovley^14^) and outer-membrane c-type cytochromes are considered to play a key role in external interspecies electron transfer. Later it was revealed that not only in *Geobacter* species which harbor specific e-pili, but DIET also occurred between *Geobacter* and methanogens (*Methanothrix* and *Methanosarcina*) which are not yet known to contain similar electric conduit machinery^15,16^. The addition of naturally-occurring or artificially synthesized conductive materials was found to stimulate DIET activity between *Geobacter* and selected methanogens^17–19^. In addition, it appears that chemically synthesized magnetite (Fe_3_O_4_) nanoparticles can complement the function of outer-membrane c-type cytochrome in a *Geobacter* mutant for DIET activity^20^. These studies imply that the presence of e-pili conductive structure and macromolecules may not be obligately necessary if environmental substitutes are provided. Genomic analysis, on the other hand, predicts that canonical syntrophs, like *S*. *wolfei*, do not have DIET-mediating accessories and hence presumably not capable of DIET^8,21^. However, enrichment cultivation from environmental samples demonstrated that addition of conductive magnetite accelerated butyrate oxidation and CH_4_ production^22,23^. The stimulating effect was detected in enrichments either with or without *Geobacter*. These studies point to a possibility of DIET in syntrophic butyrate oxidation. Likewise, a number of environmental studies have shown that different conductive materials including iron minerals, activated carbon, biochar, carbon cloth can stimulate methanogenic decomposition of either defined organic compounds like ethanol^24^, propionate^25,26^ and benzoate^27^ or complicated organic matter in bioreactors^28–30^. A recent study, however, showed that the presence of carbon nanotubes (CNTs) stimulated not only butyrate oxidation by a defined coculture consisting of *S*. *wolfei* and *Methanospirillum hungatei* but also CH_4_ production by methanogens in pure cultures^31^. Therefore, though the possibility of DIET in canonical syntrophy can not be rule out, conclusive results have yet to be obtained^14^.

A critical strategy for syntrophs to survive thermodynamic limitation is to lower the cost for energy-consuming electron discharging by exploring potential resources in environment. DIET, especially if conductive nanomaterials from environment are employed, is considered kinetically and economically more efficient than H_2_/formate-mediated electron transfer^25,32,33^. CNTs and reduced graphene oxide (rGO) are artificially synthesized carbon nanomaterials. Owing to their unique physicochemical properties including high electrical conductivity, superior chemical and mechanical stability, their production and application have been increased steadily in recent decades^34^. Inevitably, these nanomaterials will enter environments and eventually accumulate in sediments^35^. Release of CNTs could occur at all steps in the life cycle of consumer products, including: electronics, tires, textiles, manufacturing, fuel system components, landfills, sports equipment, windmill blades, injection molding, and incineration^36^. Release of CNTs from products can potentially occur by two pathways: (a) where free CNTs are released directly, or (b) where release of particles with CNTs embedded in the matrix^36^. We hypothesized that syntrophic butyrate oxidation in lake sediment could be enhanced by carbon nanomaterials. The purpose of the present study was to: (i) develop butyrate oxidation enrichments from lake sediments and determine the effect of CNTs addition; (ii) analyze microbial composition of enrichments to identify the organisms involved in syntrophic oxidation of butyrate in sediments; and (iii) construct a defined coculture comprising *S*. *wolfei* and *Mthanococcus maripalidus* to verify the effect of carbon nanomaterials on butyrate oxidation.

## Materials and Methods

### Preparation of CNTs, rGO and Fe_3_O_4_

Commercially available carboxylic functionalized multi-walled CNTs were purchased from Sigma-Aldrich (755125, USA). The rGO was purchased from Chengdu Organic Chemicals Co. (Chinese Academy of Sciences). According to the manufacturers, the average diameter and length of CNTs were about 9.5 nm and 1.5 μm, respectively, and the COOH content was about 8% (w/w). Stock water suspension of 0.49% rGO (w/v) was used for the experiment. Fe_3_O_4_ nanoparticles were synthesized by slowly adding Fe(II)/Fe(III) acidic solution (0.8 M FeCl_3_ and 0.4 M FeCl_2_ in 0.4 N HCl) into vigorously mixed 1.5 N NaOH solution^37^.

### Lake sediments and enrichment cultivation

Sediment samples were collected from the Weiming lake (WM in short), an urban lake located in the campus of Peking University (39°59′36″N 116°18′12″E), and the Erhai lake (EH in short), a natural lake located in the Yungui plateau within Yunnan Province in the southwestern China (26°01′N 100°03′E). WM has an area of about 2 ha and an average water depth of 1 m, which freezes temporally in winter. The sediment sample was characterized with the total organic carbon (TOC) of 6.25%, total nitrogen (TN) of 0.38%, and pH (H_2_O) 7.78. The EH is the second largest freshwater lake (256.5 km^2^) in the Yungui plateau, has an altitude of 1934 m and an average water depth of 10.5 m. The water source of EH is rainfall and ice-snow melt water. The sediment was characterized with TOC of 2.40%, TN of 0.27% and pH (H_2_O) 7.20. Sediment samples from both lakes were collected at the depth of 0–15 cm from the sediment surface by a 3-liter sampler.

The HEPES-buffered (30 mM, pH 7) anaerobic basal medium was used for the enrichment cultivation. Preparation of the basal medium followed the protocol described previously^38^. The cysteine was excluded in the medium to avoid the possible effect of electron shuttle molecules^39^. Sodium butyrate was added as substrate at the final concentration of 10 mM through injection via an aseptic syringe into the bottled culture medium.

The scheme for enrichment cultivation was depicted in the Fig. S1. For the first transfer, approximately 0.5 g (WM) or 5 g (EH) of fresh sediments were transferred into sterile 120 ml serum bottles filled with 40 ml of basal medium. The WM sediment has a higher TOC content than the EH sediment, and hence, a higher inoculum ratio was applied to EH enrichment cultivation in the first transfer. Incubation bottles were closed with butyl rubber stoppers and flushed with N_2_/CO_2_ [80:20 (V/V)] for 5 min.

Four continuous transfers were conducted for enrichment cultivation. For every transfer, triplicate incubations were prepared in parallel with (final concentration of 5 g L^−1^ CNTs) and without CNTs (CK). Inoculants were taken from the last enrichment with CNTs (Fig. S1). During the third and fourth transfers, a separate batch of incubations were prepared with the addition of Fe_3_O_4_ (the final concentration of 10 mM in Fe atom) in replacement of CNTs. For all transfers (except the first), the inoculum size was 4% (v/v) and enrichments were incubated statically in the dark at 30 °C under the atmosphere of N_2_/CO_2_ [80:20 (V/V)].

The cultures from the fourth transfer were subjected to microscopy and molecular phylogenetic analyses (see below). The concentrations of butyrate and acetate were also analyzed for this transfer incubations.

### Cultivation of the defined coculture

*Syntrophomonas wolfei* (DSM102351) and *Methanococcus maripaludis* (DSM14266) were purchased from German culture collection DSMZ (Braunschweig, Germany). The *S. wolfei* was cultivated in medium containing 20 mM sodium crotonate as described previously^40^. The *M. mariplaudis* was cultivated in a modified DSMZ141 medium containing 100 mM NaCl, 7.87 mM MgCl_2_.6H_2_O and 0.007 mM Fe(NH_4_)_2_(SO_4_)_2_. In addition, *M. maripaludis* was routinely grown on 170 kPa of H_2_/CO_2_ (80:20, v/v).

Coculture of *S. wolfei* and *M. mariplaudis* was initiated with a 10% inoculum of each partner organism grown to mid-logarithmic. The medium for coculture was the same with the medium for *M. mariplaudis* described above, but with the addition of 10 mM sodium butyrate. The headspace was pressurized with 170 kPa of N_2_/CO_2_ (80:20, v/v) for coculture. All the bottles were sealed with butyl stoppers and crimped aluminum caps, and the incubation temperature for cultivations was set at 35 °C.

The effect of carbon nanomaterials on the defined coculture of *S. wolfei* with *M. mariplaudis* were tested with the addition of CNTs and rGO at the final concentration of 2 g L^−1^ and 0.1 g L^−1^, respectively. Methane production by pure culture *M. maripaludis* were also investigated in the presence of carbon nanomaterials at a concentration range from 0.2 g L^−1^ to 5 g L^−1^ for CNTs and from 0.02 g L^−1^ to 0.2 g L^−1^ for rGO, respectively.

### Chemical analyses

The CH_4_ concentration was analyzed using a gas chromatograph (7890, Agilent Technologies, USA) equipped with flame ionization detector (FID). Gas samples (100 μl) were collected from the headspace using a Pressure-Lok precision analytical syringe (Bation Rouge, LA USA) every 2 to 4 days. The unit of CH_4_ concentration was converted from partial pressure in headspace to mmol L^−1^ in liquid medium by using the Avogadro’s Law. Liquid samples (0.5 ml) were collected every 4 to 9 days with a sterile syringe, centrifuged, and filtered through 0.22 µm filters. Concentrations of butyrate and acetate in culture medium were determined by high performance liquid chromatography with a ZORBAX SB-Aq C18 organic acid column (250 by 4.6 mm; particle size 5 μm; Agilent) at a flow rate of 0.8 ml/min. The UV absorbance detector was set at 210 nm.

### Molecular analysis of microbial community in enrichments

The cells in the fourth transfer subjected to CNTs treatment was harvested during the mid-log phase and used to extract total DNA using the FastDNA SPIN Kit (MP Biomedicals, USA). Prior to DNA extraction, sonication treatment was performed to separate the microbial cells from CNTs. The DNA extracts from triplicate cultures were mixed and stored at −20 °C.

For constructing the bacterial and archaeal clone libraries from WM enrichment, the extracted DNA was amplified using primer sets Ba27f/907r for bacteria and Ar109f/Ar915r for archaea, respectively. PCR products were purified and clone library analyses was performed as described previously^41^. At least 100 clones were randomly selected from each library and sequenced with an 3730 × l DNA analyzer (Applied Biosystems). The clone libraries were analyzed by defining operational taxonomic unit (OTU), in which representative sequences from each OTU shared at least 97% sequence identity. The closest matching sequences in the NCBI database (https://www.ncbi.nlm.nih.gov) were searched using the BLAST program. Phylogenetic trees were constructed using MEGA 6.0 with the neighbor-joining method. The bacterial and archaeal 16S rRNA gene sequences determined in this study were deposited in GenBank databases under accession numbers from KU743990 to KU743993.

For the EH enrichment, the high throughput Miseq sequencing was used for the analysis of microbial community. The V3-V4 universal primers 314 F/805 R were used for bacterial 16S rRNA gene amplification^42^. The archaeal 16S rRNA genes were amplified using the primer sets of 349F/806R^43^. Sequencing were performed using Illumina Miseq 2 × 300 bp platform (California, USA) by Sangon Biotech Company (Shanghai, China). More than 30, 000 sequences were obtained from each sample. The high quality sequences were processed to generate OTUs at 97% sequence similarity threshold as previously described^44^. The Ribosomal Database Project (RDP) classifier (http://rdp.cme.msu.edu) was used to assign the taxonomic data to the representative sequences^45^. Raw sequencing reads have been deposited into the NCBI Sequence Read Archive (SRA) with the accession number SRP068809 and SRP068811.

### Microscopy analysis

The cell slurries in the mid-log phase from both the enrichments and the defined coculture were collected using a sterile syringe. Fluorescence *in situ* hybridization (FISH) analysis was performed on 4% paraformaldehyde-fixed samples according to a procedure described elsewhere^46^. Oligonucleotide probes specific for bacteria (Cy3-labeled EUB338mix probes) and archaea (FITC-labeled ARC915 probe) were used in this study. The details of the probes used are available in the probeBase (http://probebase.csb.univie.ac.at/)^47^. The labeled samples were visualized using epifluorescence microscopy (Axio imager D2, ZEISS).

For scanning electron microscopy (SEM) analysis, cell slurries were fixed with 2.5% (v/v) glutaradehyde in phosphate-buffered saline, sequentially dehydrated with serial ethanol dilutions (20, 40, 60, 80, 95 and 100% (v/v) with every 10 min per step). The dried samples were coated with platinum and imaged using scanning electron microscope (FEI NanoSEM 430).

## Results

### Sediment enrichments

**Figure 1:**
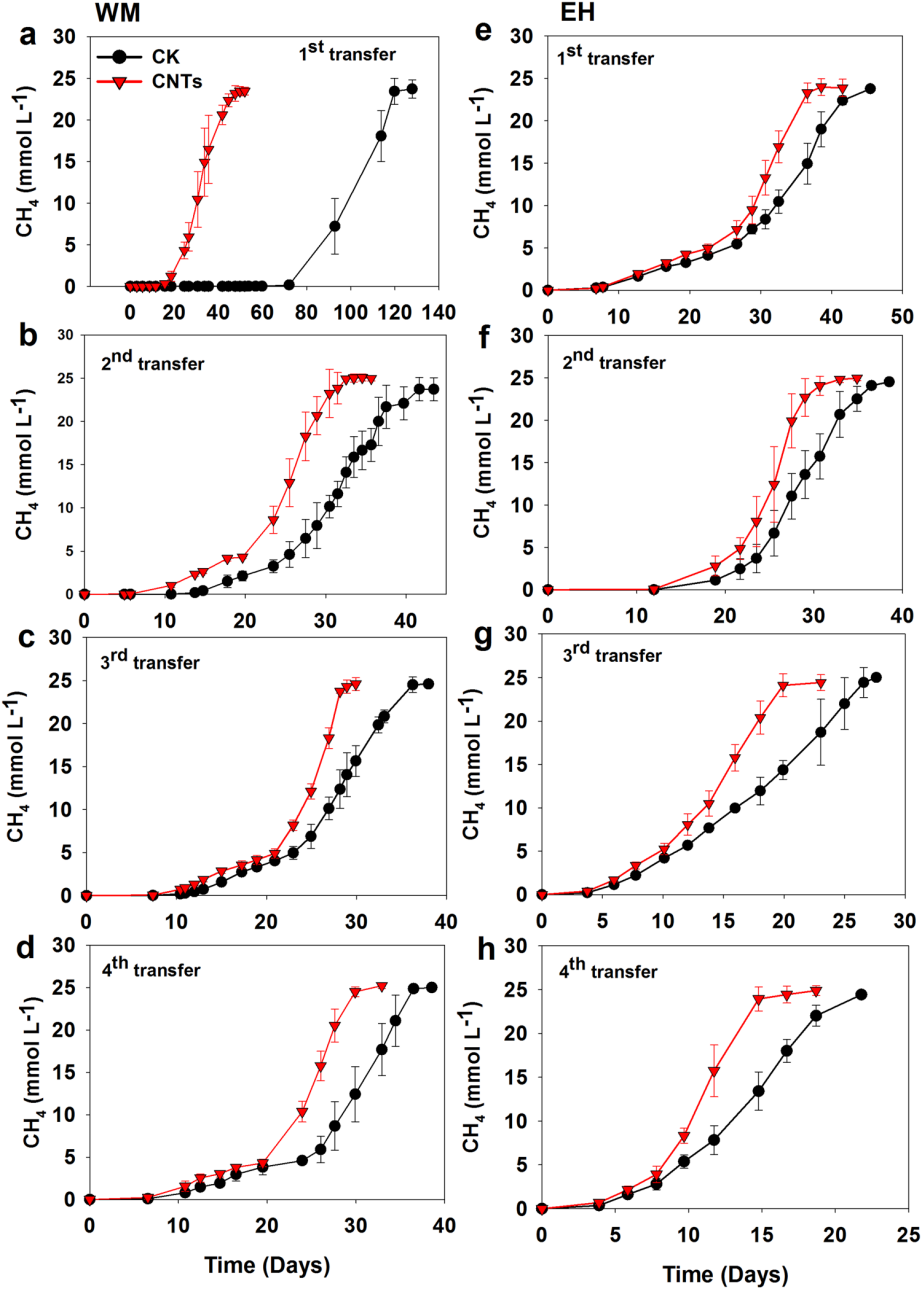
Effects of CNTs supplementation on the CH_4_ production in the enrichments from WM (**a**–**d**) and EH (**e**–**h**). Error bars represent the standard deviation of three replicates.

Two enrichments were developed from lake sediment. Addition of 10 mM butyrate produced approximately 25 mM of CH_4_ (normalized to liquid phase). Acetate accumulated transiently and eventually decreased to an undetectable level. Thus, butyrate oxidation followed the stoichiometric conversion of butyrate to CH_4_ and CO_2_. EH enrichment showed a shorter lag phase before the onset of rapid CH_4_ production (Fig. 1a,b), possibly due to the larger inoculant volume for the first transfer compared with WM enrichment. In all four transfers for both WM and EH enrichments, CH_4_ production was substantially accelerated with addition of CNTs compared with the control (Fig. 1). The maximum rate calculated based on CH_4_ increase during exponential phase showed an increase by 40 to 67% in WM enrichment and 38 to 102% in EH enrichment in the presence of CNTs compared with the control (Fig. S2).

**Figure 2:**
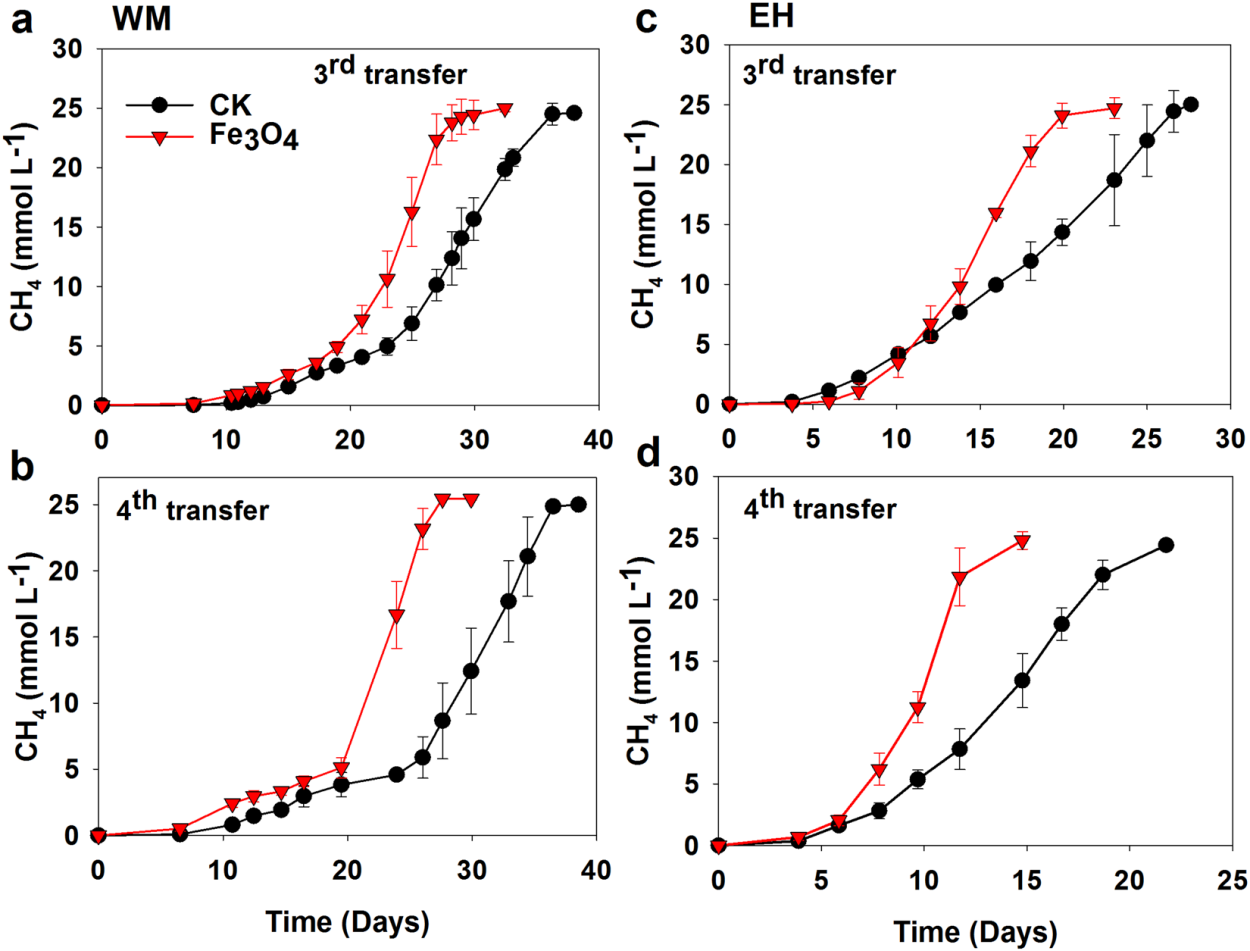
Effects of conductive nanoFe_3_O_4_ on the CH_4_ production in the third and fourth transfers from WM (**a**,**b**) and EH (**c**,**d**). Error bars represent the standard deviation of three replicates.

To verify if electric conductivity played a role in the positive effect, magnetite nanoparticles (nanoFe_3_O_4_) was added for replacement of CNTs. Inoculants from the third and fourth transfer enrichment with CNTs were used. Addition of nanoFe_3_O_4_ resulted in stimulating effect similarly as CNTs in all incubations (Fig. 2). The maximum rate of CH_4_ production increased approximately by 50% in WM enrichment and 90% in EH enrichment with nanoFe_3_O_4_ compared to the control (Fig. S3).

In coincidence with CH_4_ production, the rate of butyrate depletion was significantly faster in the presence of CNTs or nanoFe_3_O_4_ than the control (Fig. S4). Transient accumulation and decomposition of acetate coincided with butyrate depletion and CH_4_ production. The faster consumption of butyrate in EH enrichment relative to WM enrichment was in agreement with its faster onset of CH_4_ production.

**Figure 3:**
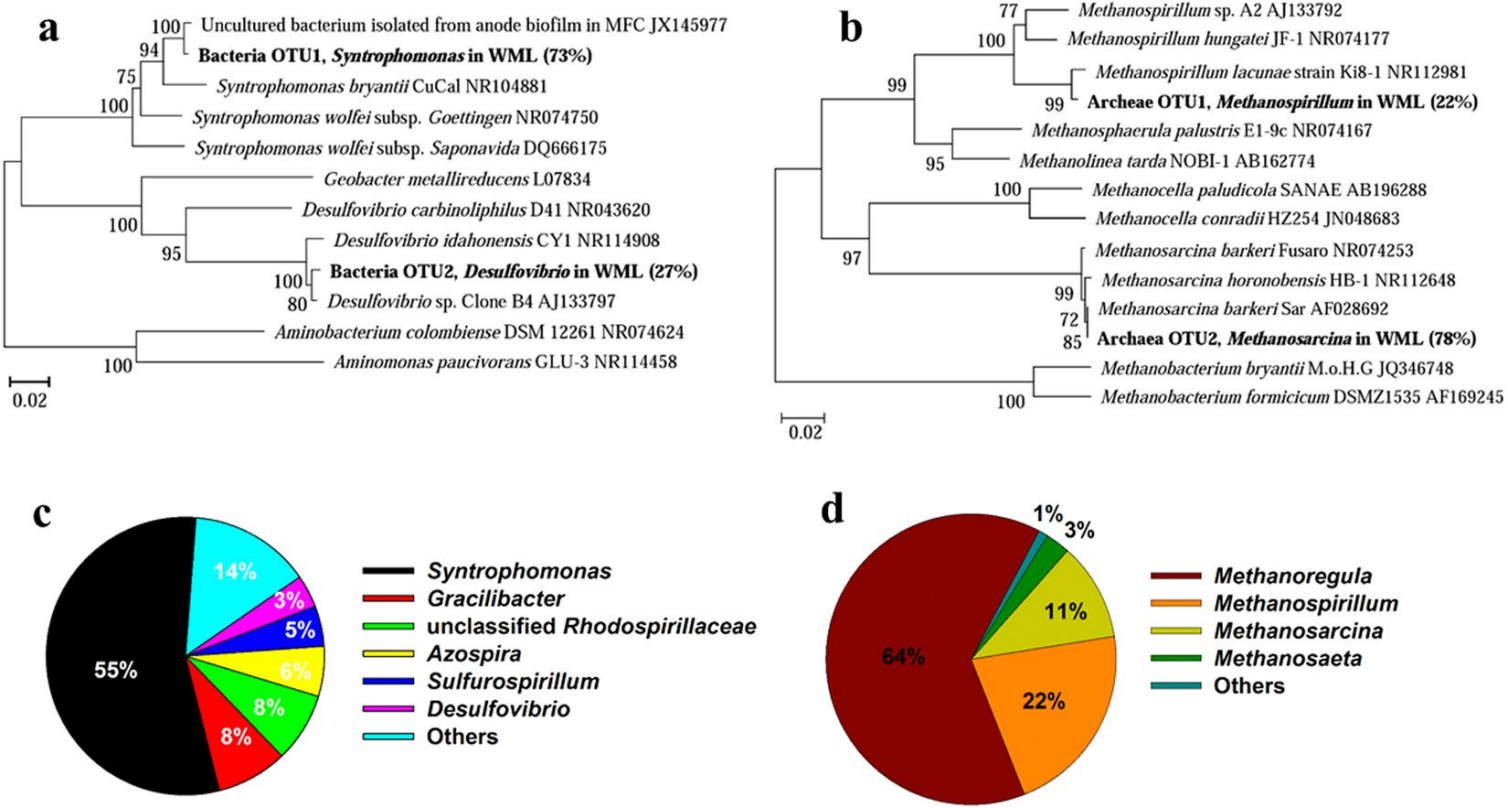
Microbial communities in the fourth transfer with CNTs addition were measured by the Sanger cloning/sequencing for WM enrichment (**a**,**b**) and high throughput Miseq sequencing for EH enrichment (**c**,**d**). Neighbor-joining phylogenetic tree of representative bacterial (**a**) and archaeal (**b**) 16S rRNA gene clones in WM enrichment. Clones obtained in this study are indicated in boldface and their relative abundances are given in parentheses (100 bacteria clones and 100 archaea clones). GenBank accession numbers of reference sequences are indicated. In addition, the phylogenetic classification and relative abundance of bacteria (**c**) and archaea (**d**) at genus level as determined by Illumina Miseq sequencing in the EH enrichment. The genus whose relative abundance was less than 2% was summarized in the group of “other” genus.

The structure of microbial community was analyzed using Sanger cloning/sequencing for WM enrichment and high throughput Miseq sequencing for EH enrichment. Miseq was used because the microbial community in EH enrichment was relatively complex based on microscopic observation (see below). One hundred clones each for bacterial and archaeal libraries were sequenced for WM enrichment. The bacterial sequences were classified into two OTUs (Fig. 3a). OTU1 accounted for 73% of total sequences, with the closest relative (99% similarity of 16S rRNA) being an uncultured bacterium isolated from the anode biofilm in microbial fuel cells (clone BP, JX145977)^48^. The closest axenic strain is *Syntrophomonas bryantii* CuCal (NR104881) sharing 95% identity of 16S rRNA sequence. OTU2, representing the rest sequences, was related to *Desulfovibrio*, with the closest relative (99% similarity) being *Desulfovibrio* sp. Clone B4 from a methanogenic enrichment culture on hexadecane^49^. The archaeal sequences also comprised two OTUs (Fig. 3b), accounting for 78% (OTU2) and 22% (OTU1) of 100 sequences, respectively. OTU2 was affiliated to *Methanosarcina*, with the closest relative (99.9% identity) of *Methanosarcina barkeri* strain Sar isolated from paddy soil^50^. OTU1 was related to *Methanospirillum* spp. The pyrosequencing of EH enrichment clone libraries revealed that the bacterial populations were dominated by *Syntrophomonas* (55%), followed by *Gracilibacter* (8%), unclassified *Rhodospirillaceae* (8%), *Azospira* (6%) and a few *Sulfurospirillum* and *Desulfovibrio* (Fig. 3c). The archaeal community comprised mainly *Methanoregula* (64%), *Methanospirillum* (22%), *Methanosarcina* (11%), and a few *Methanosaeta* (Fig. 3d).

**Figure 4:**
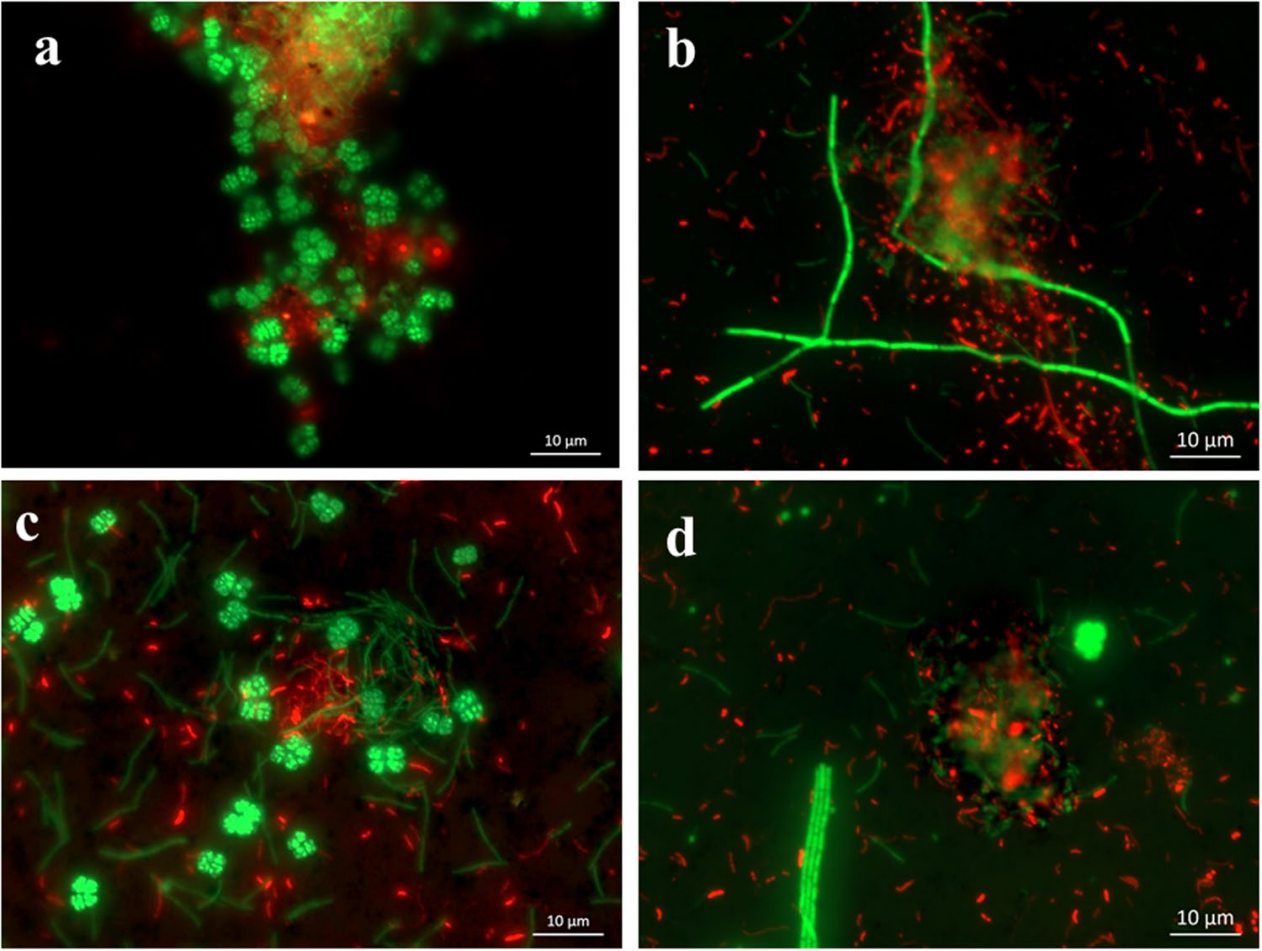
Spatial distribution of archaeal (Arc915-FITC, green) and bacterial (EUB338mix-Cy3, red) cells identified by FISH in the WM and EH enrichments. (**a**) CK of WM enrichment; (**b**) CK in EH enrichment; (**c**) CNTs treatment in WM enrichment; (**d**) CNTs treatment in EH enrichment). The CNTs concentration is 5 g L^−1^.

**Figure 5:**
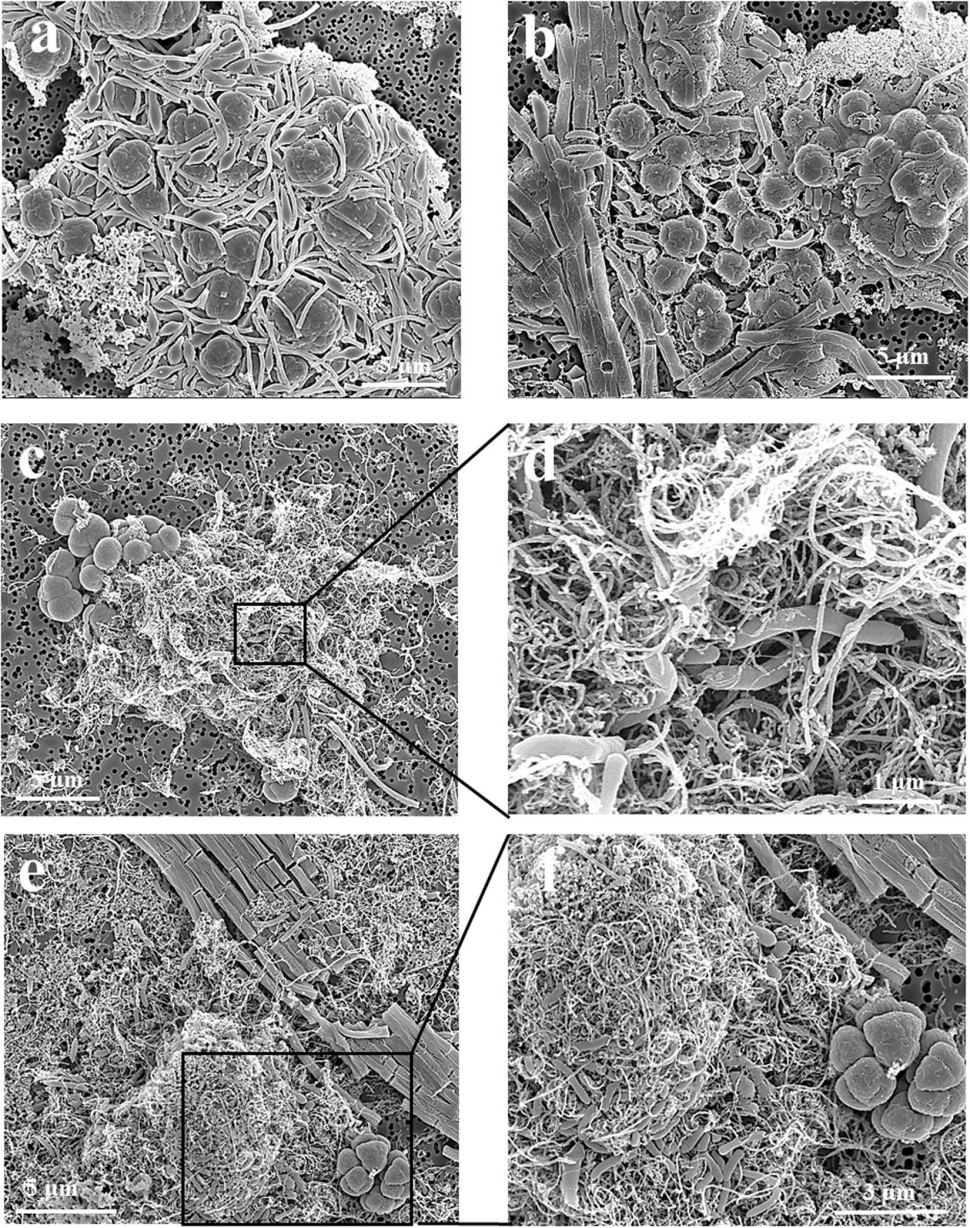
Scanning electron micrographs (SEM) images of cell aggregates in WM and EH enrichments with CK and CNTs treatments. (**a**) CK of WM enrichment; (**b**) CK in EH enrichment; (**c**,**d**) CNTs treatment in WM enrichment; (**e**,**f**) CNTs treatment in EH enrichment).

FISH and SEM assays were used to investigate the spatial organization of microbial populations and their interactions with nanomaterials. The FISH images exhibited strong fluorescence signatures of bacterial and archaeal cells in both enrichments, indicating that the cells in CNTs treatment were active and intact as in the control (Fig. 4). Most of bacterial cells exhibited coccus and short-rod shapes in enrichments. Morphology of archaeal cells was distinct between WM and EH enrichments. In the WM enrichment, classical sarcina-like shapes were dominant with slender-rod cells accounting for a small fraction (Fig. 4a,c). In the EH enrichment, long-chain, filamentous and slender-rod shaped cells were detected (Fig. 4b,d). Sarcina and slender-rod cell morphologies are indicative of *Methanosarcina* and *Methanosaeta*, respectively. FISH revealed that numerous aggregates were formed in both enrichments. The bacterial cells generally occupied the center of aggregates with archaeal cells located peripherally. However The cells within aggregates appeared less compacted (or more scattering) in CNTs treatment than in CK (Fig. 4c,d relative to Fig. 4a,b). In support of FISH observation, SEM images revealed cell morphologies of short-curve rod, slender rod with blunt ends, and sarcina-like cells (Fig. 5). The bacterial and archaeal cells in the control were in close contact forming dense microbial aggregates (Fig. 5a,b). In CNTs treatment, however, most of cells were in association with CNTs forming cells-nanomaterial mixtures (Fig. 5c–f). In addition, the SEM images showed that the cells in CNTs treatment were intact and maintaining their outer membrane structure, similar to cells in control and nanoFe_3_O_4_ treatments (Fig. S5). These results indicated that no obvious cell damage occurred when the cells were in contact with CNTs.

### Defined coculture

**Figure 6:**
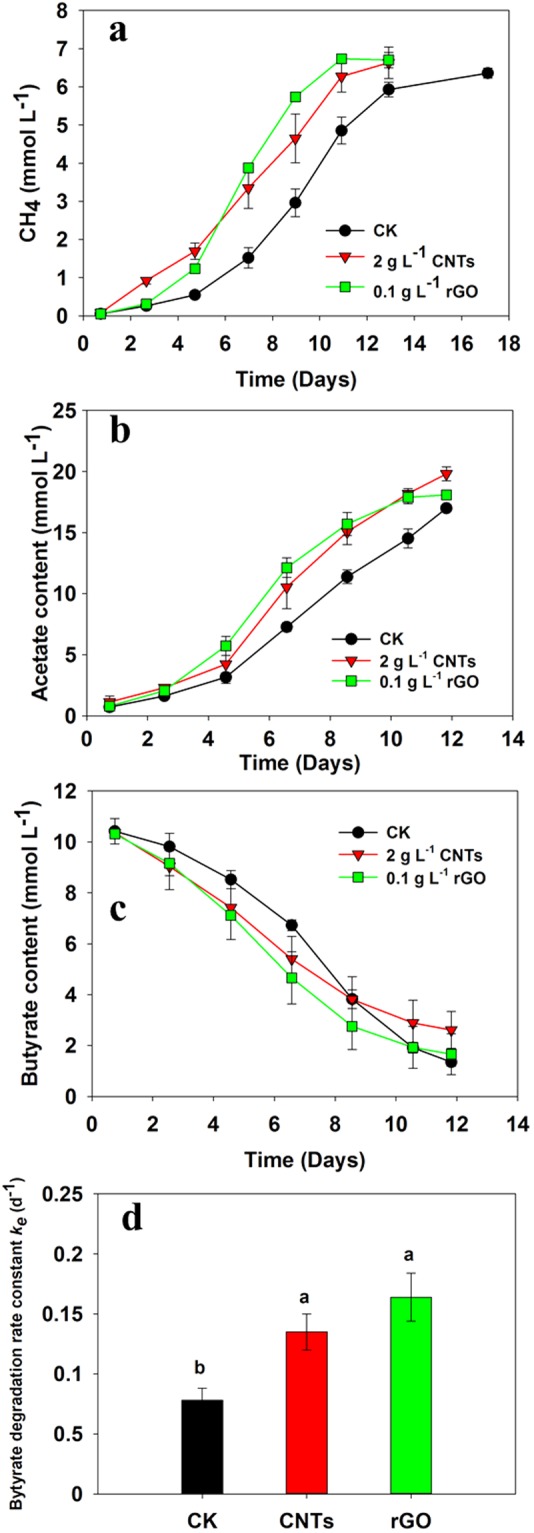
Effects of the addition of CNTs and rGO on the CH_4_ production (**a**) and acetate production (**b**), butyrate degradation (**c**) and butyrate degradation rate (**d**) in the defined coculture of *S*. *wolfei* with *M*. *mariplaudis*. Results are the mean and standard deviation for triplicate incubations. Different letters indicate significant differences (Duncan’s test, P < 0.05).

Stable coculture of *S*. *wolfei* and *M*. *maripaludis* was established after a few continuous transfers, which revealed a generation time of 57 h, shorter than the coculture of *S*. *wolfei* with *M*. *Hungatei* (84 h) reported before^51^. The degradation of 10 mM butyrate yielded about 5 mM CH_4_ (normalized to liquid phase) and 20 mM acetate in medium, indicating near to stoichiometric conversion of butyrate to CH_4_ and acetate in coculture. Two carbon nanomaterials, CNTs and rGO, were used for test. The production of CH_4_ was significantly promoted by CNTs and rGO compared with the control (Fig. 6a). Consistently, acetate accumulation and butyrate depletion were faster in CNTs and rGO than in the control (Fig. 6b,c). The rate of butyrate consumption calculated according to the first-order kinetic model increased by approximately 62% and 112% in the treatments of CNTs and rGO, respectively (Fig. 6d). To verify the effect of electric conductivity of nanomaterials, kaolinite was added to coculture at the same concentration of CNTs. Kaolinite is a clay mineral, widespread in nature but electrically nonconductive^52^. The results shown that the production of CH_4_ was not affected by the addition of kaolinite (Fig. S6). To assess if physicochemical adsorption of substrates happened with CNTs and rGO, adsorption experiment was conducted by adding butyrate and acetate to sterile medium in the presence of CNTs and rGO. The concentration of butyrate and acetate in medium remained constant over 15 days of incubation (Fig. S7), indicating no significant adsorption by carbon nanomaterials.

**Figure 7:**
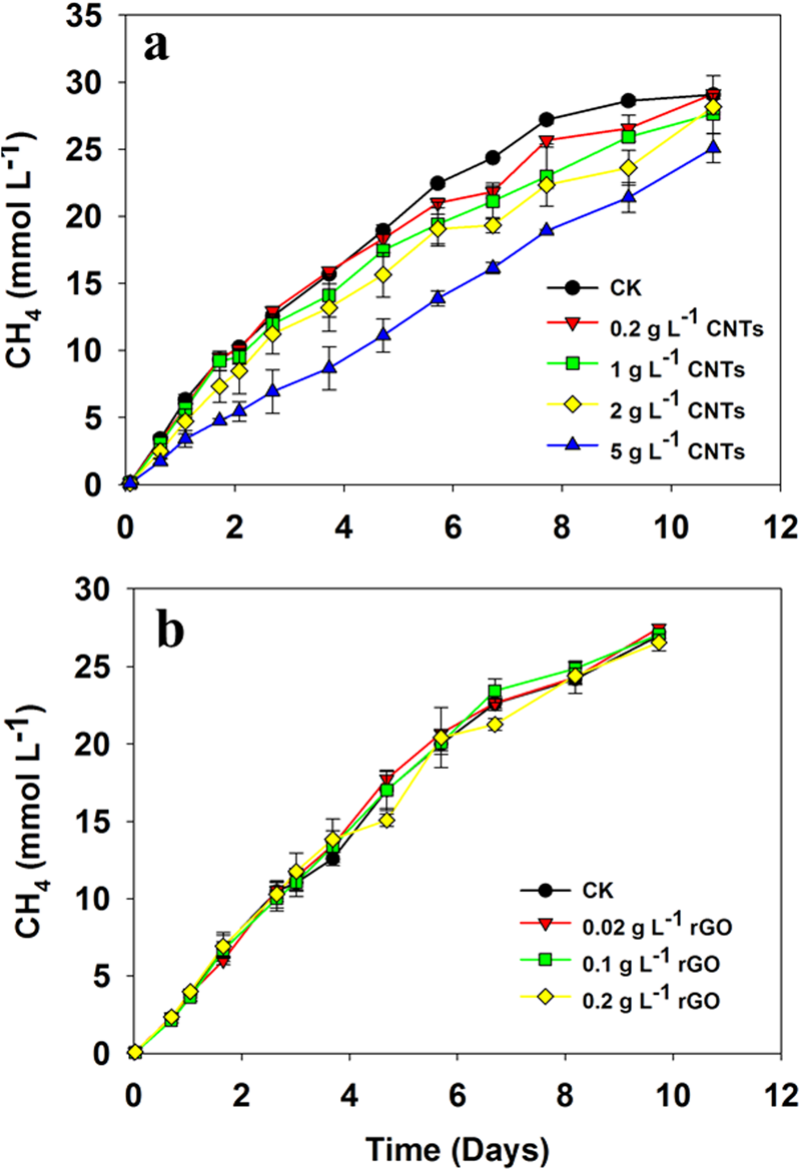
Cumulative CH_4_ production by *M. mariplaudis* in the control assays without CNTs/rGO (CK), and with addition of different concentrations of CNTs (**a**) and rGO (**b**). Results are the mean and standard deviation for triplicate incubations.

To verify if CNTs and rGO caused stimulating effect on methanogens in pure culture as suggested before^31^, a concentration gradient experiment on CNTs and rGO was performed using pure culture of *M*. *Maripaludis* with H_2_/CO_2_ (80:20, 1.7 kPa) as substrate. For a comparison, the concentration of carbon nanomaterials used in coculture experiment was 2 g L^−1^ for CNTs and 0.1 g L^−1^ for rGO, respectively. It appeared that the rate of CH_4_ production slightly decreased with the increase of CNTs from 0.2 g L^−1^ to 2 g L^−1^ and further increase of CNTs to 5 g L^−1^ substantially depressed CH_4_ production in pure culture (Fig. 7a). Addition of rGO at the concentration from 0.02 to 0.2 g L^−1^ did not show distinguishable effect on CH_4_ production (Fig. 7b).

**Figure 8:**
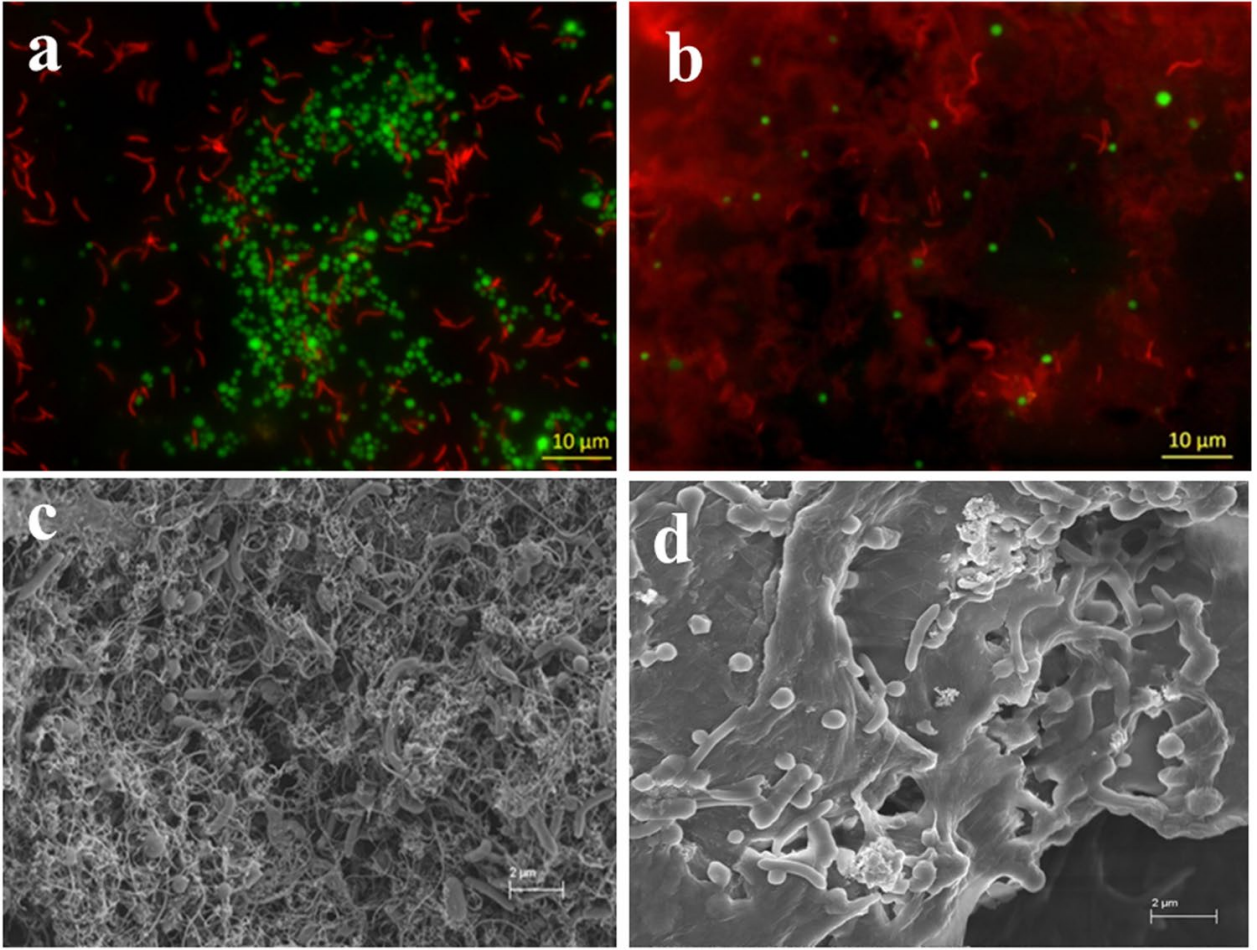
FISH (**a**,**b**) and SEM (**c**,**d**) images of the defined coculture of *S*. *wolfei* with *M*. *mariplaudis* in the presence of CNTs (**a**,**c**) and rGO (**b**,**d**). The concentrations of CNTs and rGO are 2 g L^−1^ and 0.1 g L^−1^, respectively. For the FISH images, red color indicates bacterial cell and green color indicates archaeal cell.

FISH and SEM showed distinct images between CNTs and rGO treatments. Most cells of *S*. *wolfei* (in red) and *M*. *maripaludis* (in green) in CNTs treatment displayed strong fluorescence signature (Fig. 8a), similar to the sediment enrichments described above (Fig. 4). Cells formed mixture aggregates together with CNTs (indicated by dark areas within aggregates). By comparison, the number of cells was substantially fewer in the FISH image for the rGO treatment (Fig. 8b). This result, however, did not indicate the loss of living cells, because the activity of butyrate oxidation was even higher in rGO than in CNTs treatment (Fig. 6d). A close look of FISH image revealed that many cells were actually buried under or wrapped up by the thin rGO sheets (Fig. 8b). In consistence with FISH observation, SEM image revealed the formation of microbial aggregates in CNTs treatment in which the slightly helical rod (*S*. *wolfei*) and coccus (*M*. *maripaludis*) cells were interwoven with carbon nanotubes (Fig. 8c). In the rGO treatment, cells appear scattered and adhered to the surface of thin graphene sheets with many cells buried under or wrapped up by the smooth thin rGO sheets (Fig. 8d).

## Discussion

Syntrophs represent a group of metabolic specialists, utilizing limited substrates, mainly intermediate products from anaerobic decomposition of organic matter in natural habitats^2^. How syntrophic organisms interact with environment factors, i.e. extracellular processes, however has remained poorly investigated. In the present study, we investigated the effect of carbon nanomaterials on syntrophic oxidation of butyrate in two lake sediment enrichments and a defined coculture. The results reveal that carbon nanomaterials substantially promote butyrate oxidation and imply that the nanomaterial-induced DIET is possible to occur.

Enrichment cultivation of two lake sediments reveals that butyrate oxidation and CH_4_ production was significantly promoted by CNTs right from the first incubation and in all subsequent transfers (Fig. 1). When CNTs was replaced by nanoFe_3_O_4_, similar stimulating effect was detected in both enrichments (Fig. 2). Apart from sharing common property of electric conductivity, CNTs and nanoFe_3_O_4_ are chemically different. These results suggest that conductivity of nanomaterials likely plays a key role in stimulating butyrate oxidation in the enrichments, which is in line with our previous observation on the effect of nanoFe_3_O_4_^22,23^.

The presence of different organisms in enrichments prevents an explicit explanation of positive effect on syntrophic partners. Therefore, a coculture comprising *S*. *wolfei* and *M*. *maripaludis* was constructed and tested for the effect of two carbon nanomaterials, CNTs and rGO. Both materials showed stimulating effect on butyrate oxidation (Fig. 6). A control using kaolinite, a clay mineral that is electrically nonconductive but otherwise can provide physical support for cell attachment and nutrient adsorption, revealed no effect. We further tested the effect of CNTs on M. *maripaludis* in pure culture to verify if the positive effect was due to methanogen partner as suggested previously^31^. The rGO in the concentrations range from 0.02 to 0.2 g L^−1^ revealed no effect while CNTs exerted a negative effect, especially when the concentration increased to 5 g L^−1^ CNTs (Fig. 7). Apparently, the effect of carbon nanomaterials on methanogen partner could not explain the stimulation on coculture in the present study.

CNTs have been reported to cause different inhibitory effects on microorganisms^35,53,54^. Notably, based on the pure culture test, the concentration of CNTs used for enrichment cultivation (5 g L^−1^ CNTs) and for the defined coculture (2 g L^−1^ CNTs) was already at the upper limit where negative effect on methanogen partners might take place. However, we are unable to clarify if the negative effect occurs under enrichment and coculture conditions. SEM observation revealed no obvious physical damage on microbial cells (Figs 5 and 8), and moreover, promotion rather than repression on CH_4_ production by CNTs were observed in all enrichment and coculture incubations. Previous study revealed uncertain effect of CNTs on different methanogens^31^. CH_4_ production by *Methanobacterium formicicum* was promoted steadily up to a concentration of 5 g L^−1^ CNTs. But inhibitory effect was evidenced at 5 g L^−1^ CNTs on two aceticlastic methanogens *Methanosaeta concilii* and *Methanosarcina mazei*^31^. Therefore, the effect of CNTs on methanogens in pure culture appears depending on CNTs concentration and methanogen identity. Nevertheless, if negative effect on methanogen partner took place, the stimulating effect on syntrophic metabolism in the present experiment would have been underestimated. Further investigation is necessary to delineate the effect of carbon nanomaterials on individual methanogens and anaerobic bacteria.

*Syntrophomonas* dominated the bacterial populations after four transfers in both WM and EH enrichments (Fig. 3). *Syntrophomonas* in WM enrichment shared 99% identity of 16S rRNA sequences to an uncultured bacterial clone retrieved from anode biofilm of a microbial fuel cell fed with butyrate and propionate^48^. *Geobacter* was not detected. Sequences related to *Desulfovibrio* in WM enrichment and to *Gracilibacter*, *Rhodospirillaceae*, *Azospira*, *Sulfurospirillum* and *Desulfovibrio* in EH enrichment were detected. But these bacterial lineages are not known to perform butyrate oxidation and extracellular electron transfer. Though lacking of genes encoding for the *Geobacter*-like conduit machinery in *Syntrophomonas* app.^8^, a few lines of previous evidences underscored DIET in butyrate oxidation in the presence of conductive materials or electrodes. The studies on microbial fuel cells and microbial electrolysis cells showed that *Syntrophomonas* was detected in anodic biofilms, and together with *Geobacter* could result in electricity generation from butyrate oxidation^48^. We showed previously that syntrophic oxidation of butyrate and CH_4_ production in rice paddy soil and lake sediment enrichments were enhanced by nanoFe_3_O_4_ which was likely related to DIET induced by the conductive mineral^22,23^. For the methanogen partners, increasing evidences suggest that DIET is indeed possible with certain methanogens. *Methanothrix harundinacea* that can not grow on H_2_ in pure culture can grow syntrophically with *Geobacter metallireducens*^16^. Functional genes of *M*. *harundinacea* and *G*. *metallireducens* were found to be actively transcribed in rice field soil indicating the DIET-driven CH_4_ production by *M*. *harundinacea*^55^. *M*. *barkeri* can also establish coculture with *G*. *metallireducens* and interact each other via DIET^15^. Electrochemical studies revealed that an uncharacterized *Methanobacterium*-like marine isolate was capable of utilizing electrons from cathode at redox potential above the threshold for abiotic H_2_ production^56^. Several other methanogens such as *M*. *maripaludis*^57,58^, *Methanobacterium palustre*^59^ and *Methanothermobacter* spp.^60^ have been reported to thrive in different electrochemical systems with the possibility of receiving electrons from cathodic electrodes. More of indirect evidences emerge from undefined methanogenic systems. For instance, *Methanoregula* were highly enriched (53%) in an electrical-anaerobic digestion reactor^61^. Supplementation of granular activated carbon increased the rate of CH_4_ production in continuous flow anaerobic reactor with significant enrichment of *Geobacter* and hydrogenotrophic methanogens- *Methanospirillum* and *Methanolinea*^62^.

Though decisive conclusion can not be made, our study tends to support the possibility of DIET in syntrophic oxidation of butyrate. Machinery for H_2_ and formate-mediated interspecies electron transfer has been well described^7,8^. Cumulating evidences now indicate that syntrophs use H_2_ and formate pathways simultaneously or separately depending on environmental conditions^9^. While H_2_ and formate pathways are not repulsive each other, we hypothesize that a third pathway, DIET, can work in concert with H_2_/formate pathways to cope with environmental changes. Electrochemical studies have indicated that *M*. *maripaludis* utilize electrons directly or indirectly from cathodic electrodes^57,58^. The indirect pathway, considered to be more probable, was assumed due to the release of hydrogenases from lysed cells (dead or living) and then attached to electrode surface^58^. The hydrogenases receive electrons from electrode to produce H_2_ which is then used by methanogens. This idea suggests that extracellular hydrogenases are likely to shuttle electrons between methanogen and electrode. Both externally-oriented hydrogenase and formate dehydrogenase are present in *S*. *wolfei* and Methanogens. The electron discharging from these membrane-bound hydrogenases in *S*. *wolfei* is the thermodynamically most difficult step in butyrate metabolism^7,10^. In the presence of highly conductive nanomaterials, electron transfer from externally-oriented hydrogenase to nanomaterial and vise versa can be conceived as like between electrode and hydrogenase. Our FISH and SEM images showed that bacteria and archaea cells in the enrichments and defined coculture were interwoven by CNTs forming microbial cell-CNT mixture aggregates (Figs 4, 5 and 8). For the rGO treatment, the cells of both *S*. *wolfei* and *M*. *maripaludis* were attached to, buried under, or wrapped up by the very thin graphene sheets (Fig. 8). While this spatial arrangement appears to separate the interacting cells and increase the diffusive barrier for interspecies H_2_ and/or formate transfers, the high conductivity of nanomaterials can provide effective conduit for DIET in butyrate oxidation.

In conclusion, the present study demonstrated the supplementation of carbon nanomaterials resulted in a substantial stimulatory effect on syntrophic butyrate oxidation and CH_4_ production in lake sediment enrichments and a defined coculture. Discharging electrons with minimum energy cost is the rule in canonical syntrophic metabolism. DIET is considered kinetically and economically more efficient compared with H_2_/formate-mediated electron transfer^25,32,33^. Albeit the lacking of e-pili-like structures and outer-membrane cytochromes, a provision of externally conductive nanomaterials may set a substitution opportunity for the syntrophy organisms. With the increasing manufacturing and application of carbon nanomaterials, the results of present study shall also draw an attention to the probable effect of nanomaterials on degradation of organic matter and methanogenesis in anoxic habitats that play important role in global CH_4_ emission.

## Electronic supplementary material

Supporting Materials
