# Supplementary material for: Stimulation of carbon nanomaterials on syntrophic oxidation of butyrate in sediment enrichments and a defined coculture
Source: Sci Rep. 2018 Aug 15;8:12185. doi: 10.1038/s41598-018-30745-7 (PMC6093938; doi:10.1038/s41598-018-30745-7)
Supplement: Supplementary file 1 — Supporting Materials [file 41598_2018_30745_MOESM1_ESM.pdf]

Supplementary Information

Stimulation of carbon nanomaterials on syntrophic oxidation of butyrate in sediment enrichments and a defined coculture

Wei Zhang[a] *, Jianchao Zhang[b] *, Yahai Lu[a]

(*Contribute equally to this work)

For correspondence: YH Lu, College of Urban and Environmental Sciences, Peking University, No. 5 Yiheyuan Road, Beijing 100871, China. E-mail luyh@pku.edu.cn; Tel. +86 10 62755683; Fax +86 10 62755683.

Captions of Supporting Figures

Figure S1: Schematic diagram of the experiment design with different treatments and transfers. For the transfers (except the first transfer), the inoculum size was 4% (v/v), and the inoculum was from the culture with CNTs treatment. The incubations were carried out in triplicate.

Figure S2: Effects of CNTs on the maximum methanogenic rates in WM (a) and EH (b) enrichments.

Figure S3: Effects of nanoFe_3O_4 on the maximum methanogenic rates in the third and fourth transfers in WM (a) and EH (b).

Figure S4: Butyrate oxidation and acetate formation in the fourth transfer of enrichment cultures with or without nanomaterials (CNTs and nanoFe_3O_4) supplementation from WM (a) and EH (b).

Figure S5: SEM images of cells-nanoFe_3O_4 aggregates in the WM (a) and EH (b) enrichments. White arrows indicate Fe_3O_4 nanoparticles.

Figure S6: Effects of kaolinite on the CH_4 production from butyrate degradation in the defined coculture of *S. wolfei* with *M. mariplaudis.*

Figure S7: Butyrate (a) and acetate (b) adsorption experiments for the CNTs and rGO in the sterile medium.

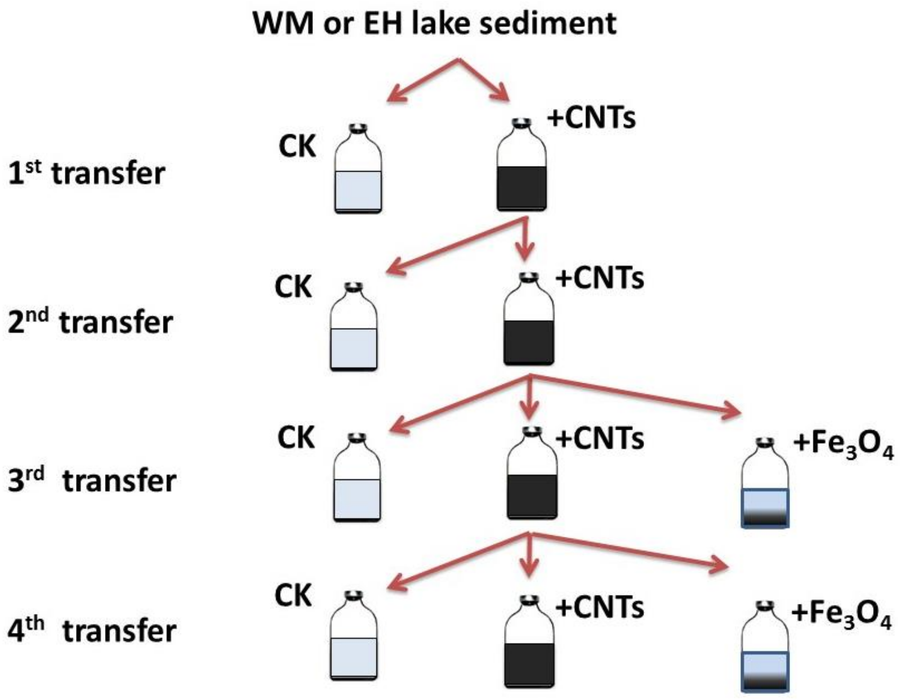

Figure S1 Schematic diagram of the experiment design with different treatments and transfers. For the transfers (except the first transfer), the inoculum size was 4% (v/v), and the inoculum was from the culture with CNTs treatment. The incubations were carried out in triplicate.

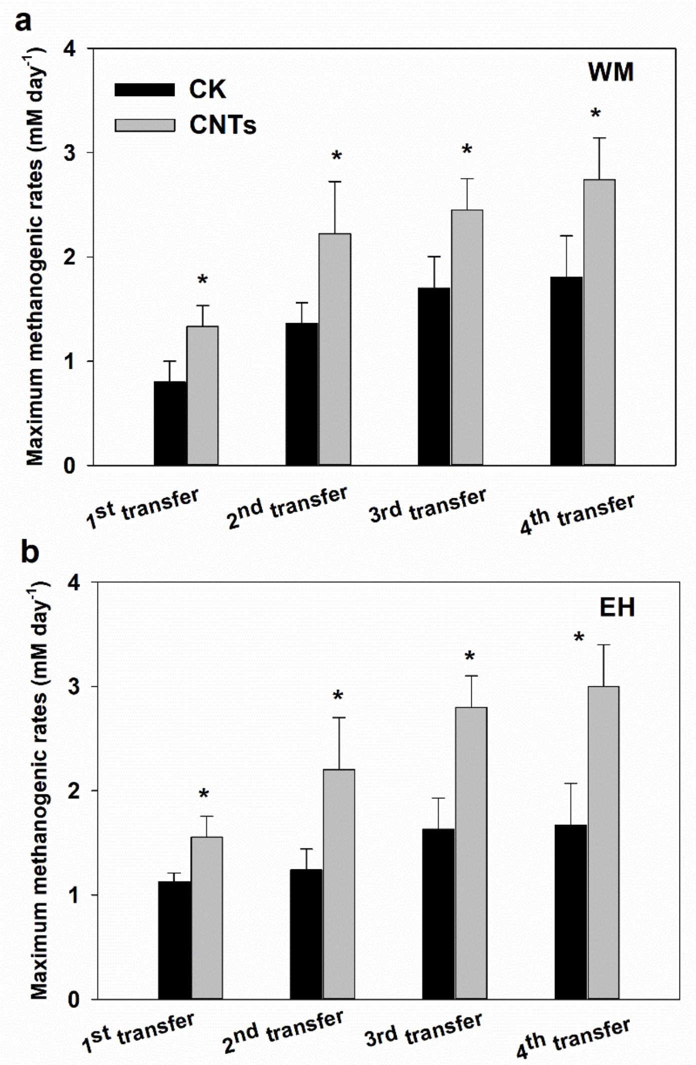

Figure S2 Effects of CNTs on the maximum methanogenic rates in WM (a) and EH (b) enrichments. Asterisks represent a significant difference ($P < 0.05$) between treatments with Student's t test.

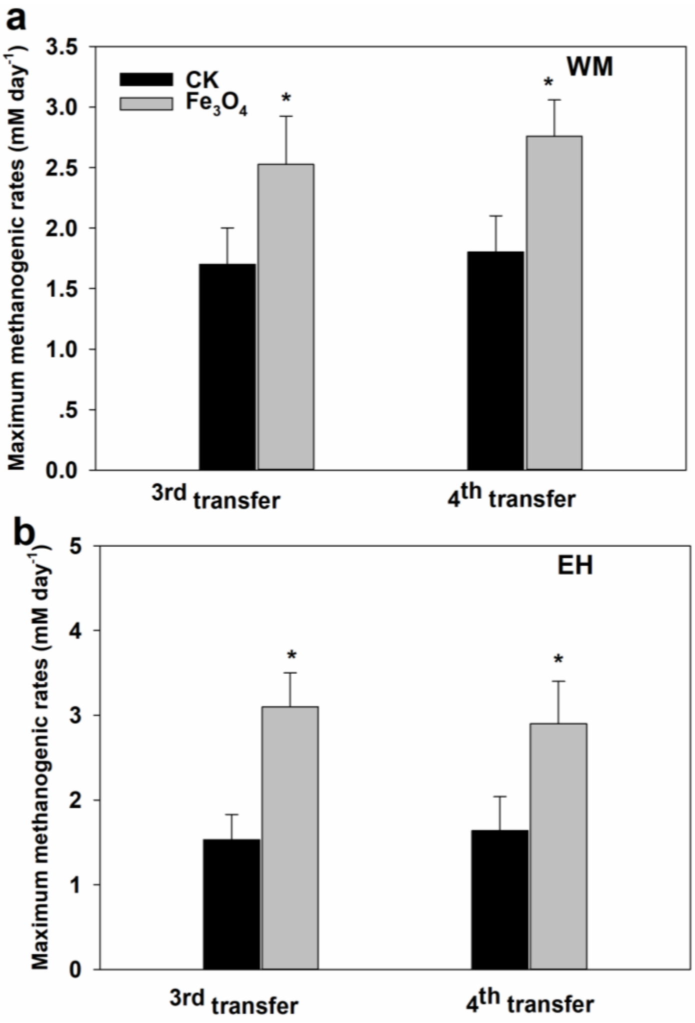

Figure S3 Effects of nanoFe_3O_4 on the maximum methanogenic rates in the third and fourth transfers in WM (a) and EH (b). Asterisks represent a significant difference ($P < 0.05$) between treatments with Student's t test.

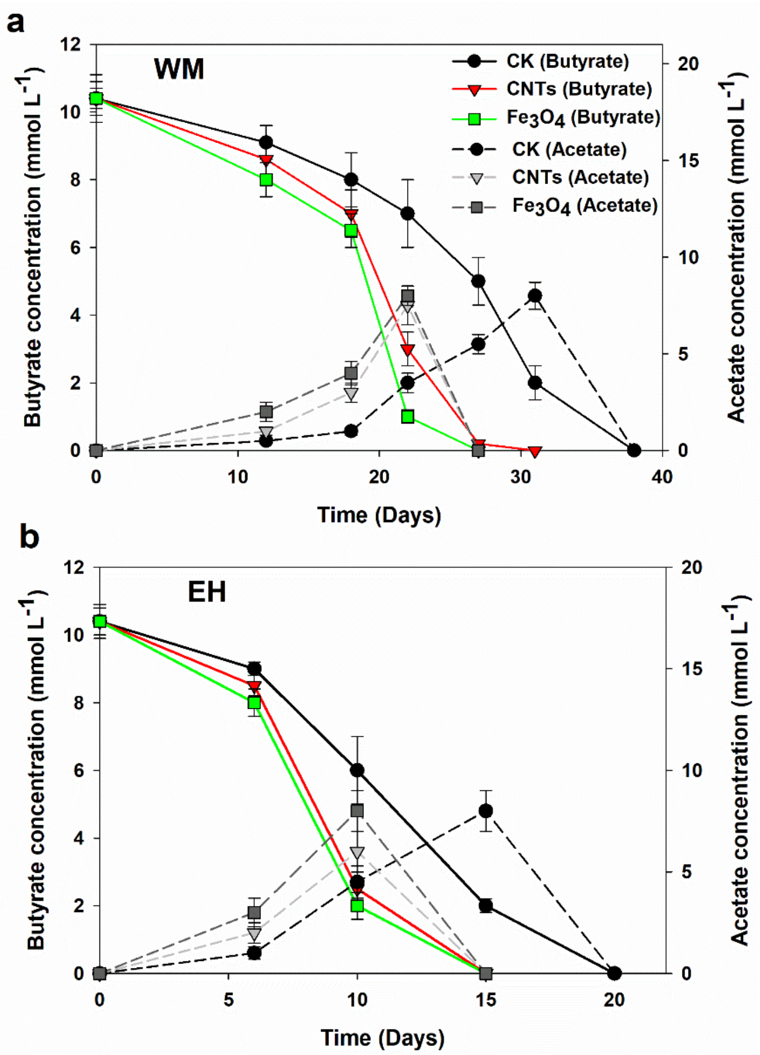

Figure S4 Butyrate oxidation and acetate formation in the fourth transfer of enrichment cultures with or without nanomaterials (CNTs and nanoFe_3O_4) supplementation from WM (a) and EH (b).

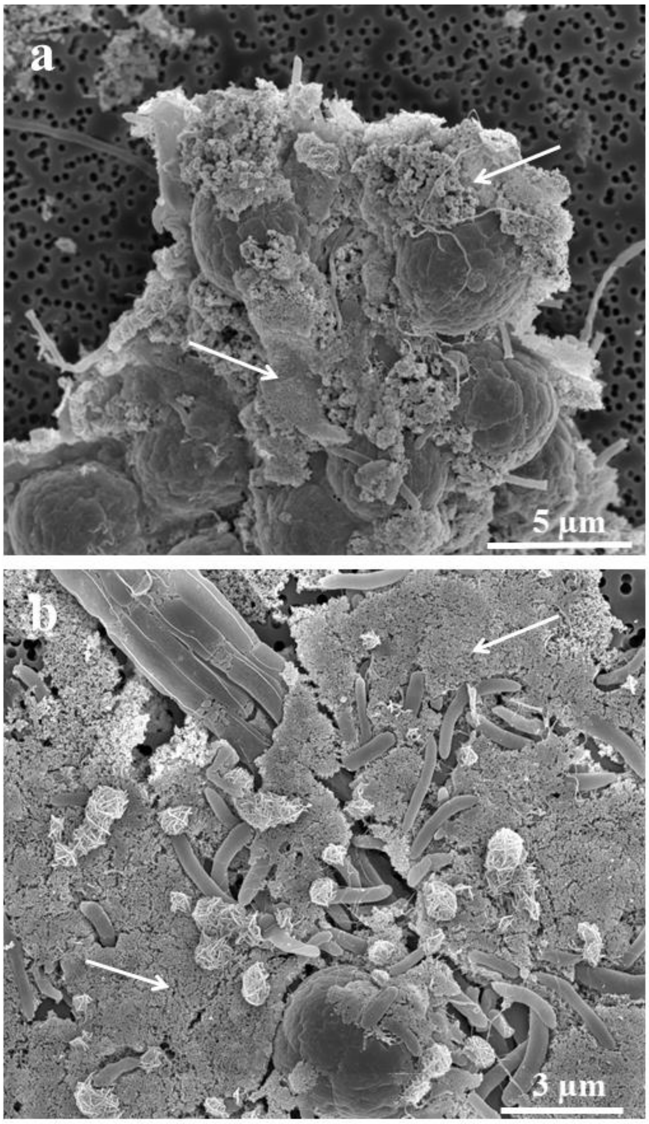

Figure S5 SEM images of cells-nanoFe_3O_4 aggregates in the WM (a) and EH (b) enrichments. White arrows indicate Fe_3O_4 nanoparticles.

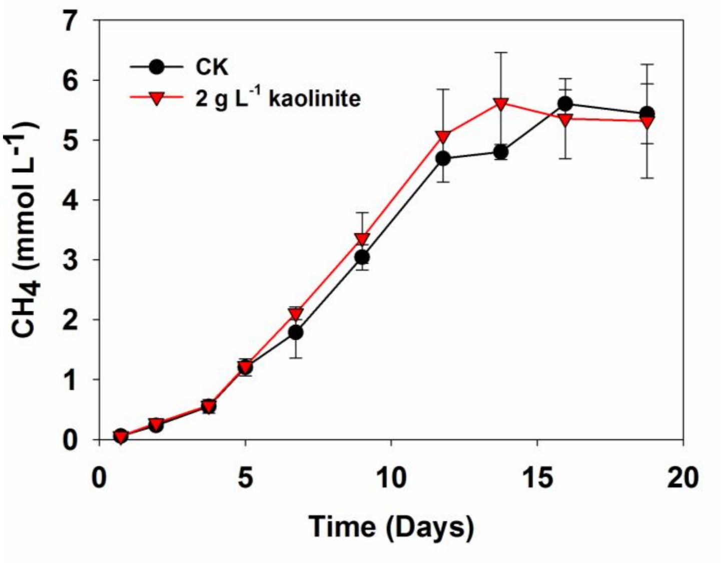

Figure S6 Effects of kaolinite on the CH_4 production from butyrate degradation in the defined coculture of *S. wolfei* with *M. mariplaudis.*

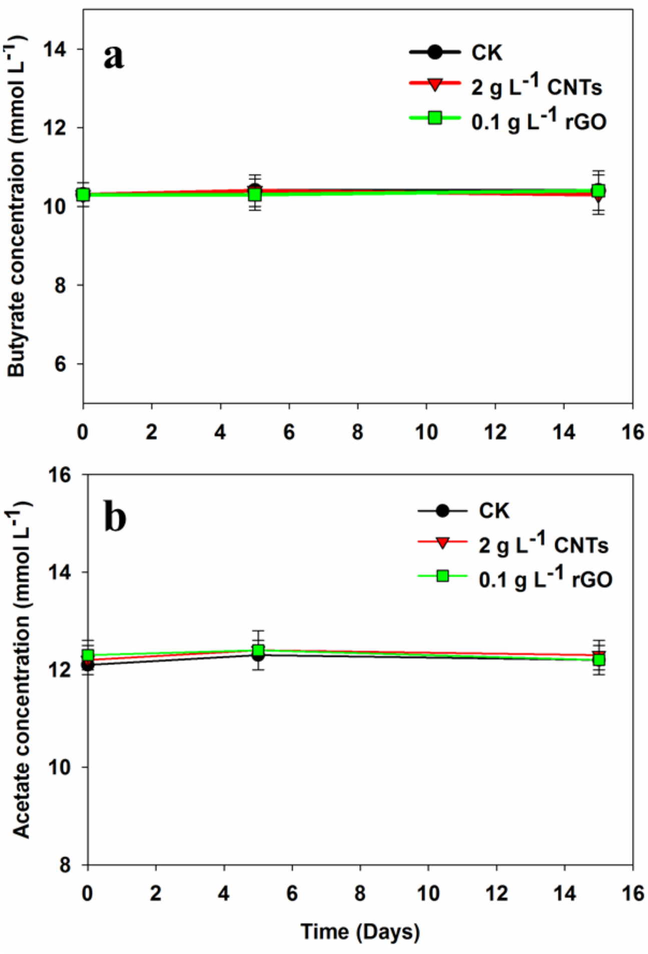

Figure S7 Butyrate (a) and acetate (b) adsorption experiments for the CNTs and rGO in the sterile medium.
